# Clinical Accuracy of an Intraoral Scanner for Tooth Shade Selection Compared With a Spectrophotometer and Visual Matching: A Comparative Study

**DOI:** 10.1002/cre2.70347

**Published:** 2026-03-31

**Authors:** Amr Sameer Baqleh, Nabil Alhouri

**Affiliations:** ^1^ Department of Fixed Prosthodontics, Faculty of Dentistry Damascus University Damascus Syria

**Keywords:** intraoral scanner, shade matching, spectrophotometer, value

## Abstract

**Objectives:**

This study evaluated the performance of an intraoral scanner (Medit i700) for tooth shade selection by comparing it with a spectrophotometric reference device (VITA Easyshade) and visual observers, using VITA Classical and VITA 3D‐Master shade guides.

**Material and Methods:**

Sixteen patients (48 right maxillary teeth: central incisor, canine, and first molar) were recruited from the fixed prosthodontics clinic. For each tooth, shade was recorded three times with Medit i700, three visual observers, and VITA Easyshade under standardized daylight conditions using both VITA Classical and VITA 3D‐Master guides. Easyshade was used as the reference. Exact shade matching between each method and the reference was calculated as percentages. A secondary analysis focused on the value (brightness) component, assessing exact value match and value acceptability within ± 1 value level. Chi‐square tests were applied to explore the influence of method, shade guide, and tooth type on value outcomes (*α* = 0.05).

**Results:**

Exact shade matching was low for all methods and both shade guides. When only value was considered, agreement increased substantially: the intraoral scanner and visual observers achieved moderate exact value matches and high value acceptability (typically > 80% within ± 1 value level of the reference) across both shade guides. Chi‐square analysis showed no significant differences in value performance between methods; however, shade guide type showed a significant effect on exact value matching, while value acceptability did not differ. In contrast, tooth type had a statistically significant influence on value outcomes, with higher agreement in molars (and, to a lesser extent, canines) than in incisors (*p* < 0.05).

**Conclusions:**

Within the limitations of this clinical study, the intraoral scanner Medit i700 did not provide sufficient accuracy for full shade selection, but showed high agreement for value determination. Intraoral scanners should be integrated with spectrophotometric and controlled visual assessment for shade matching in esthetic restorations.

## 1 Introduction

Shade selection is a critical step in fixed and esthetic dentistry, as the visual integration of the restoration with the adjacent teeth strongly influences patient satisfaction and perceived treatment success. Conventional shade selection is most commonly performed visually, using shade guides such as VITA Classical and VITA 3D‐Master. However, visual shade matching is inherently subjective and may be affected by the observer's experience, fatigue, background conditions, and the illumination environment, which can result in considerable inter‐ and intra‐observer variability in clinical practice (Paul et al. 2002; Paravina et al. 2002). Therefore, standardization of viewing conditions and examiner calibration is essential in comparative shade studies.

To reduce the influence of human factors, various electronic systems have been introduced, most notably spectrophotometers such as the VITA Easyshade. These devices analyze the spectral distribution of reflected light and convert it into a corresponding tooth shade in a standardized color space, and several clinical studies have reported higher repeatability and accuracy for spectrophotometric measurements compared with visual shade selection (Vildósola et al. 2017). These advantages have been consistently confirmed in later clinical studies (Liberato et al. 2019; Ebeid et al. 2020; Yassin 2019). For this reason, Easyshade has often been used as a reference instrument in in vivo research on tooth color determination. In the present study, Easyshade was used as a clinical reference instrument due to its established repeatability.

In parallel with the digitalization of restorative workflows, modern intraoral scanners (IOSs) have incorporated shade‐measurement functions that allow simultaneous capture of three‐dimensional geometry and tooth color. Systems such as Medit i700, TRIOS, and other high‐resolution IOS have been evaluated for shade matching in comparison with spectrophotometers and conventional visual methods, with most studies reporting that IOS provide more stable results than visual matching but generally do not reach the same level of agreement as spectrophotometry (Brandt et al. 2017; Culic et al. 2018). Comparable outcomes have been reported in more recent investigations using different IOS systems (Czigola et al. 2021; Floriani et al. 2024; Lee et al. 2024). Additional clinical studies have supported these findings across various tooth types and shade guides (R. Akl et al. 2022; Valente et al. 2020). Nevertheless, the clinical performance of the shade‐matching function remains a topic of ongoing debate, especially in routine prosthodontic settings (M. Akl et al. 2023).

Another important aspect of tooth color determination is the role of the Value (brightness) component. Experimental and clinical work in dental color science has indicated that discrepancies in Value are less acceptable to patients than differences in hue or chroma, and that small deviations in brightness can have a marked impact on esthetic outcome (Paravina et al. 2004; Ragain and Johnston 2000). Despite this, many clinical studies on IOS shade matching have focused on overall shade agreement only, without specifically quantifying Value matching or the proportion of cases falling within a clinically acceptable range of brightness.

Moreover, tooth type and shade‐guide design may further influence the accuracy of shade determination. Anterior teeth, particularly maxillary incisors, exhibit higher translucency and more complex optical behavior, which can complicate shade assessment, whereas posterior teeth with more convex surfaces tend to show more uniform reflection patterns (Culic et al. 2018; Yoon et al. 2016).

In addition, the VITA 3D‐Master system provides a more structured numerical organization of shades, prioritizing Value and chroma gradation compared with the traditional VITA Classical arrangement, which may facilitate digital shade comparison in some contexts (Paravina et al. 2004).

The aim of this study is to evaluate the accuracy of the Medit i700 IOS in tooth shade selection compared with the VITA Easyshade spectrophotometer and visual shade matching by three trained observers, with VITA Classical and VITA 3D‐Master shade guides. The primary outcome was the proportion of exact shade matching to the spectrophotometric reference. Secondary outcomes included exact Value agreement and Value acceptability within ± 1 value level. The null hypotheses were that (1) there would be no significant differences in shade or Value matching between methods, (2) shade guide type would not significantly influence matching performance, and (3) tooth type (incisor, canine, AND molar) would not significantly affect the outcomes.

## 2 Materials and Methods

### 2.1 Study Design

This comparative clinical study was conducted at the Department of Fixed Prosthodontics, Faculty of Dentistry, Damascus University. A total of 16 patients (48 teeth), aged 20–35 years, medically and orally healthy, were recruited.

Selected teeth: (1) Maxillary right central incisor, (2) Maxillary right canine, and (3) maxillary right first molar. This selection represented different anatomical and optical characteristics across the dental arch.

- Inclusion criteria: Vital, intact teeth without caries, restorations, cracks, or discoloration, no bleaching within the last 6 months, healthy periodontal tissues.
- Exclusion criteria: Endodontically treated or restored teeth, orthodontic appliances, developmental defects, enamel opacities, or severe wear, systemic conditions affecting enamel.

The study protocol was reviewed and approved by the Research Ethics Committee of the Faculty of Dentistry, Damascus University, Damascus, Syria. The ethics committee does not assign a specific protocol number; approval was granted based on the official committee meeting held on January 18, 2025. Official written ethical approval documentation is available upon request. All procedures were conducted in accordance with the ethical standards of the institutional research committee and with the Declaration of Helsinki. Written informed consent was obtained from all participants before their inclusion in the study.

### 2.2 Examiner Selection and Calibration (Pilot Study)

Three visual examiners were selected from postgraduate residents. The number three was intentionally chosen to reduce individual bias and increase the reliability of visual assessment.

**Figure 1:**
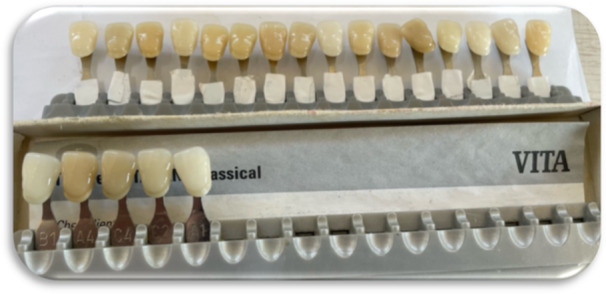
Shade guides used for calibration of visual examiners during the pilot study. Five reference shade tabs (A1, B1, A4, C2, and C4) from a VITA Classical shade guide were used for the calibration task. The shade codes of the second guide were masked, allowing examiners to match each reference tab with the corresponding tab under standardized lighting conditions.

For calibration, two identical VITA Classical shade guides were used. From the first guide, five shade tabs were selected to represent a range of easy (A1, B1) and difficult (A4, C2, and C4) shades. These five tabs were left fully visible with their shade codes. On the second VITA Classical guide, all shade codes were covered with an opaque strip, while keeping the labial surfaces of the tabs completely visible. Under standardized lighting conditions, each candidate was asked to match each of the five reference tabs from the first guide with its corresponding tab on the masked second guide. One point was awarded for each correct match, yielding a score from 0 to 5. Ten postgraduate students were tested, and the three candidates with the highest scores (4 correct matches out of 5, which was the best performance obtained) were selected as the visual examiners for the main study. This calibration procedure ensured a consistent and standardized level of visual shade‐matching ability among the selected examiners (Figure 1). Agreement among the three examiners during the pilot matching task was evaluated descriptively to confirm consistency in shade identification.

### 2.3 Lighting Conditions

All assessments were performed under natural daylight between 11:00 a.m. and 2:00 p.m. in the same clinical area to reduce variation in illumination. No artificial lighting was used. Patients were seated upright with the occlusal plane parallel to the floor, and shade evaluation was performed within approximately 5 s to minimize eye fatigue and color adaptation. A neutral clinical background was maintained to reduce visual bias.

### 2.4 Shade‐Matching Protocol

Teeth were cleaned with a soft brush and water spray without polishing paste to avoid modifying surface reflectivity. Shade determination was performed at the middle third of the labial surface for the central incisor and canine. For the first molar, the measurement was taken on the middle third of the buccal surface. The same anatomical area was used for all three repetitions and across all methods. Each tooth underwent three repeated measurements with each method in the following fixed order: (1) IOS: The Medit i700 shade function was used according to the manufacturer's workflow. Shade was recorded from the same predefined tooth area after scanning. (2) Visual shade matching (three calibrated examiners): Each visual examiner was allowed a maximum of 5 s per observation to determine the shade in order to minimize retinal fatigue. Shade evaluation was performed at an approximate distance of 25–30 cm from the tooth. A short neutral gaze interval between observations was allowed to reduce visual adaptation. (3) Spectrophotometer (VITA Easyshade 4.0 — reference): The VITA Easyshade 4.0 was calibrated according to the manufacturer's instructions before each patient. The probe tip was positioned perpendicular to the tooth surface at the predefined area, ensuring stable contact and minimizing ambient light interference.

A fixed measurement sequence was used for all participants. Visual examiners were blinded to the IOS results, and the reference device (VITA Easyshade) was used after visual assessment to avoid influencing examiner choices.

**Figure 2:**
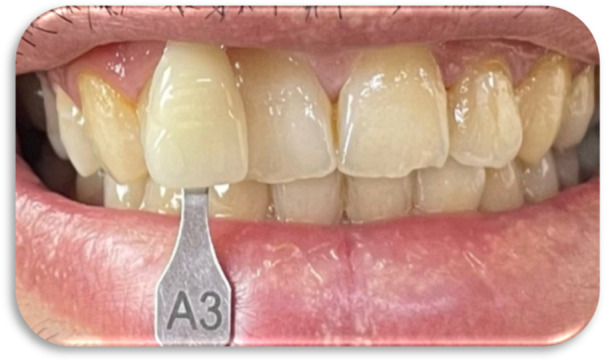
Visual tooth shade selection using the VITA Classical shade guide.

**Figure 3:**
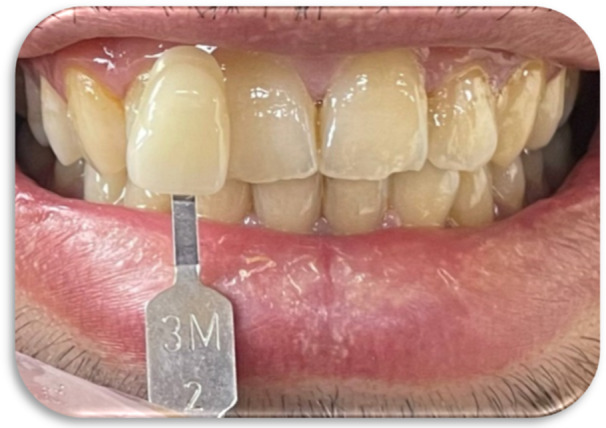
Visual tooth shade selection using the VITA 3D‐Master shade guide.

**Figure 4:**
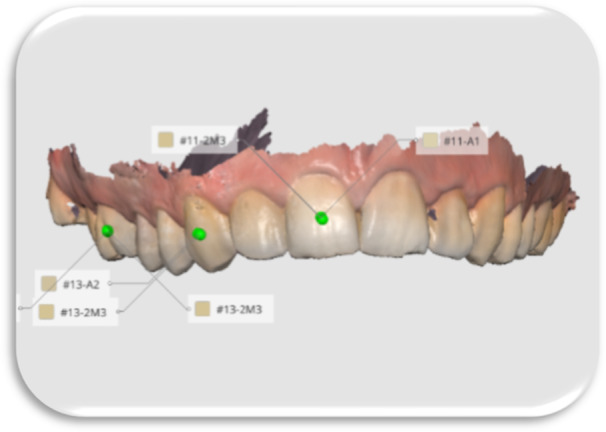
Shade determination with Medit i700 intraoral scanner.

**Figure 5:**
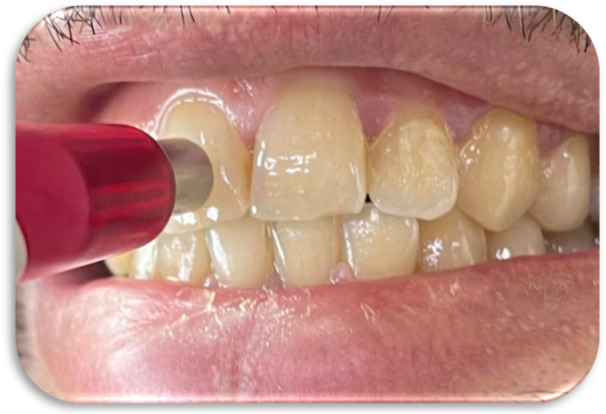
Shade determination with VITA Easyshade 4.0.

In all methods, shade selection was performed using the VITA Classical and the VITA 3D‐Master shade guides (Figures 2, 3, 4, 5).

### 2.5 Data Recording

Each method produced three repeated readings per tooth per shade guide. For each method, the final recorded shade was defined as the mode (most frequently occurring value) of the three readings. An agreement was considered present when at least two of the three measurements resulted in the same shade designation.

No “no‐mode” cases were observed; therefore, the mode was available for all recordings.

### 2.6 Statistical Analysis

Data were analyzed using SPSS version 25.0. Descriptive statistics were used to calculate percentages of exact shade matches for all methods, and a chi‐square test was used to compare differences in match frequencies between methods. The significance level was set at *α* = 0.05. The unit of analysis was the tooth–shade guide–method combination (48 teeth × 2 shade guides × 4 methods = 384 observations). Because multiple teeth were collected from each participant, observations may not be fully independent; therefore, chi‐square findings should be interpreted cautiously.

## 3 Results

### 3.1 Shade‐Matching Agreement

Exact shade matching was generally low across all methods. When VITA Classical was used, agreement with the reference device (VITA Easyshade) did not exceed 31% in incisors, 31% in canines, or 44% in molars among visual observers, while the Medit i700 achieved 31%, 19%, and 13%, respectively. Using the VITA 3D‐Master guide yielded comparable findings, with exact‐match percentages ranging between 13% and 38% across tooth types and methods. Overall, Exact shade agreement remained low across methods and shade guides (Table 1).

**Table 1:** Exact shade match using VITA Classical and VITA 3D‐Master.

Method	Classical incisor (%)	Classical canine (%)	Classical molar (%)	3D‐Master incisor (%)	3D‐Master canine (%)	3D‐Master molar (%)
Medit i700	31	19	13	13	38	38
Visual 1	31	31	13	38	25	25
Visual 2	25	0	44	19	25	38
Visual 3	19	25	19	25	31	25

### 3.2 Transition to Value‐Based Analysis

Because exact shade matching remained consistently low, a detailed review of the mismatches showed that many discrepancies involved teeth sharing similar Value levels despite differences in hue or chroma. This pattern suggested that focusing solely on the full shade matching underestimates the true degree of agreement. Therefore, subsequent analyses emphasized Value (brightness) as an independent parameter, given its established dominance in visual shade perception and its higher consistency across digital and visual methods.

### 3.3 Exact Value Match

When the analysis shifted to the Value (brightness) component, agreement increased markedly. Using VITA Classical: Medit i700 achieved exact value matches of 63% (incisors), 50% (canines), and 69% (molars). Visual observers reached values ranging from 56% to 88%, depending on tooth type.

With VITA 3D‐Master, value‐match percentages followed a similar pattern, ranging between 50% and 75% across tooth types and methods. These findings demonstrate that brightness is more consistently detected than hue/chroma across all methods (Table 2).

**Table 2:** Value match using VITA Classical and VITA 3D‐Master.

Method	Classical incisor (%)	Classical canine (%)	Classical molar (%)	3D‐Master incisor (%)	3D‐Master canine (%)	3D‐Master molar (%)
Medit i700	63	50	69	50	69	63
Visual 1	88	63	63	69	63	69
Visual 2	63	56	88	69	63	75
Visual 3	69	75	69	63	69	63

### 3.4 Value Acceptability (± 1 Value Level)

Before analyzing value outcomes, both shade guides were reorganized according to brightness levels. In the VITA 3D‐Master guide, shades are inherently arranged in five value groups (1–5). For the VITA Classical guide, each shade tab was assigned to one of five value categories by matching it to its closest corresponding 3D‐Master shade under the same lighting conditions (Table 3). Within this value‐based classification, shades belonging to the same category were considered to share the same value level, and deviations of ± 1 value level were interpreted as one step lighter or darker.

**Table 3:** Classification of VITA Classical shade guide according to value levels.

G1	A1	B1						
G2	A2	B2	D2					
G3	A3	A3.5	B3	B4	C1	C2	D3	D4
G4	A4	C3						
G5	C4							

When accepting a ± 1 level deviation in Value, agreement rose sharply to above 80%–100% across almost all combinations of method and tooth type. Medit i700 achieved: 100% agreement in incisors and canines (Classical and 3D‐Master), 81%–94% agreement in molars.

Visual observers similarly demonstrated high acceptability levels ranging between 81% and 100%. This indicates that although exact shade matching is poor, most mismatches occur within one step of Value, which aligns with established clinical thresholds of acceptability (Table 4).

**Table 4:** Value acceptability (± 1 value level) using VITA Classical and VITA 3D‐Master.

Method	Classical incisor (%)	Classical canine (%)	Classical molar (%)	3D‐Master incisor (%)	3D‐Master canine (%)	3D‐Master molar (%)
Medit i700	100	100	81	100	94	94
Visual 1	100	100	100	100	94	88
Visual 2	94	88	88	100	94	100
Visual 3	94	94	81	94	100	88

### 3.5 Effect of Assessment Method on Value

Chi‐square testing revealed no significant association between the assessment method (Medit i700 vs. the three observers) and value outcomes: exact value match, *p* = 0.888 > 0.05, and value acceptability, *p* = 0.979 > 0.05. This indicates that the scanner and visual observers performed similarly with respect to brightness determination (Table 5).

**Table 5:** Chi‐square tests for associations between study factors and value outcomes (*N* = 384).

Factor	Outcome	*N*	*χ* ^2^	df	*p*‐value
Method	Value match (exact)	384	0.632	3	0.888
	Value acceptability (± 1 level)	384	0.193	3	0.979
Shade guide	Value match (exact)	384	5.723	1	0.017
	Value acceptability (± 1 level)	384	0.193	1	0.661
Tooth type	Value match (exact)	384	7.811	2	0.020
	Value acceptability (± 1 level)	384	8.775	2	0.012

### 3.6 Effect of Shade Guide on Value

A statistically significant association was found between shade guide type and exact value match, *p* = 0.017 < 0.05, with the VITA Classical guide showing a higher proportion of exact value matches than VITA 3D‐Master. In contrast, value acceptability within ± 1 value level did not differ significantly between the two shade guides, *p* = 0.661 > 0.05. These findings suggest that while both guides behave comparably in terms of clinically acceptable value matching, VITA Classical may allow slightly more precise value matching at the exact level (Table 5).

### 3.7 Effect of Tooth Type on Value

Tooth type had a statistically significant influence on value outcomes: *p* = 0.020 < 0.05 for exact value match and *p* = 0.012 < 0.05 for value acceptability. Agreement percentages were highest in molars, followed by canines, and lowest in incisors (Table 5).

## 4 Discussion

The present clinical study evaluated the performance of an IOS (Medit i700) and three trained visual observers in reproducing the tooth shade provided by a spectrophotometric reference device (VITA Easyshade). The analysis was carried out at two levels: (1) exact shade matching according to VITA Classical and VITA 3D‐Master; (2) agreement in the Value (brightness) component, both as an exact match and within a ± 1 value level tolerance. In addition, the influence of shade guide type and tooth type on value outcomes was explored.

### 4.1 Exact Shade Matching

In line with previous clinical and in vivo investigations, exact shade matching in this study remained limited for all methods, regardless of whether VITA Classical or VITA 3D‐Master was used. Several authors have reported modest agreement when full shade codes (hue–chroma–value) from visual assessment, spectrophotometers, or IOSs are compared directly (Paul et al. 2002; Brandt et al. 2017). Similar findings have been reported in more recent clinical investigations (Liberato et al. 2019; Czigola et al. 2021), and in systematic reviews evaluating IOS accuracy in color determination (Tabatabaian et al. 2024). Comparable outcomes have also been observed in studies using contemporary IOS systems (Floriani et al. 2024; Abu‑Hossin et al. 2023). This modest performance reflects the cumulative effect of many factors, including the discrete and sometimes unbalanced distribution of shades in commercial guides, variability in illumination, surface texture, translucency of enamel, and observer fatigue. In this study, the Medit i700 could not overcome these inherent limitations. Its exact shade‐matching percentages remained clearly below an ideal clinical target. This agrees with previous work showing that IOSs, while useful for integrating color into digital workflows, are still constrained by similar shade‐guide mapping issues and optical artefacts as other devices (R. Akl et al. 2022; Rutkunas 2020). As a consequence, relying solely on the nominal shade label—whether determined visually or digitally—may underestimate the true degree of similarity between the compared shades.

### 4.2 Rationale for Focusing on Value

A detailed inspection of the mismatched cases revealed that many “disagreements” in full shade matching involved teeth that shared similar brightness levels but differed in hue and/or chroma. This observation is clinically relevant because brightness has repeatedly been shown to be the dominant component in the visual acceptability of dental color (Ragain and Johnston 2000; Johnston 1989). This concept was further reinforced by subsequent experimental and clinical studies (Paravina et al. 2004; Joiner 2004).

Small deviations in hue or chroma can often be compensated by characterization, glazing, or surrounding structures, whereas errors in value are much more visible, particularly in the maxillary anterior region.

On this basis, this study introduced a secondary analysis that isolated the Value component from the full shade code. Both VITA Classical and VITA 3D‐Master shades were regrouped into five value levels. For 3D‐Master, this followed the inherent 1–5 value structure of the guide; for Classical, shades were allocated to the corresponding levels. This allowed the shade information from different systems and methods to be compared on a common value scale.

### 4.3 Value Match and Value Acceptability

When exact value agreement was considered, the scanner and the observers achieved moderate to high percentages, typically in the 50%–70% range depending on tooth type and shade guide. These findings are in line with studies that have emphasized the relative robustness of brightness compared with hue and chroma in shade selection (Li and Wang 2007). Similar observations have been reported in clinical studies using instrumental and digital methods (Vildósola et al. 2017; Liberato et al. 2019). More recent investigations have confirmed these findings using contemporary IOS systems (R. Akl et al. 2022).

More importantly, when a ± 1 value level tolerance was allowed, value acceptability increased to ≥ 80%–100% in almost all combinations of method, tooth type, and shade guide. Psychophysical research suggests that such small differences in brightness often fall around or below perceptibility and acceptability thresholds under typical clinical conditions (Paravina et al. 2011; Sharma et al. 2005). The present data therefore indicate that, although exact shade matching may look discouraging, most mismatches in this study occurred within a narrow value range that is likely to be clinically acceptable.

From a clinical perspective, these results reinforce the importance of prioritizing value selection and considering a small tolerance range, rather than expecting full shade codes from different methods to coincide perfectly.

### 4.4 Scanner Versus Visual Observers

The chi‐square analysis showed no significant association between assessment method and value outcomes. Neither exact value matching nor value acceptability differed significantly between the Medit i700 and the three trained observers. This suggests that, under controlled conditions and when the analysis focuses on brightness, the IOS performs similarly to skilled human observers.

These findings are consistent with reports in which IOSs and spectrophotometers have not consistently outperformed experienced clinicians in all aspects of shade selection, especially when strict thresholds are used for defining agreement (Culic et al. 2018; Czigola et al. 2021). Similar trends have also been reported in more recent studies (Abu‑Hossin et al. 2023).

Rather than replacing visual judgment, digital systems should be viewed as complementary tools that provide standardization, documentation, and improved communication with dental laboratories.

### 4.5 Effect of the Shade Guide System

A significant effect of the shade guide was detected for exact value matching: VITA Classical yielded higher exact value agreement than VITA 3D‐Master, while value acceptability (± 1 level) did not differ significantly between the two guides. Both systems, once regrouped into five value levels, offered similarly high value acceptability within ± 1 level. This finding is noteworthy in light of the design philosophy of 3D‐Master. The 3D‐Master system was developed to provide a more systematic arrangement of shades based on value, chroma, and hue, and several authors have highlighted its advantages for structured shade selection and improved reproducibility when full shade codes are considered (Brandt et al. 2017; Paravina et al. 2004). The reproducibility of both shade guide systems has also been addressed in previous investigations (Hampe‑Kautz et al. 2020). However, most of those investigations analyzed overall shade accuracy or instrumental color differences (Δ*E*), whereas the present work focused on the categorical value levels as defined by the guides. One possible explanation for the better exact value performance of VITA Classical is operator familiarity. Classical remains the most widely used guide in daily practice, and clinicians may be more accustomed to its distribution of shades and to interpreting its tabs in terms of “light” and “dark,” even if its layout is less uniform from a colorimetric standpoint. In addition, within each value level, 3D‐Master includes a broader range of chroma and hue, which could increase the chance that small differences in these components lead to different nominal shade labels, even when the perceived brightness is similar. Grouping the Classical shades into five value categories allowed a direct comparison with the 3D‐Master system. This reflects how clinicians tend to cluster shades by brightness in daily practice, yet future studies could improve this value mapping by using instrumental measurements on the original shade tabs.

### 4.6 Effect of Tooth Type

Tooth type significantly influenced both exact value matching and value acceptability. Molars showed the highest value agreement with the reference device, followed by canines and incisors. This gradient is consistent with the known optical characteristics of different tooth types. Posterior teeth typically present thicker enamel and dentin layers, smaller visible areas, and reduced transparency, all of which contribute to more stable light reflection and easier value judgment. In contrast, maxillary incisors are more translucent, and their appearance is strongly affected by the underlying dentin, the oral cavity background, and surrounding soft tissues, making their brightness more sensitive to changes in viewing and lighting conditions (Culic et al. 2018; Yoon et al. 2016). Similar trends have been reported in previous studies that found greater variability and lower agreement in shade selection for anterior teeth compared with posterior teeth (Liberato et al. 2019; Floriani et al. 2024). These findings were observed regardless of whether the assessment was visual, spectrophotometric, or scanner‐based (Abu‑Hossin et al. 2023).

The present results therefore reinforce the recommendation that special attention should be paid to value control in the anterior region. Combining multiple methods, repeating measurements, and considering the contralateral tooth as a reference are particularly important when planning highly visible restorations.

### 4.7 Limitations and Future Directions

This study has several limitations that should be considered when interpreting the results. First, the sample size was relatively modest (16 patients, 48 teeth), which may limit the statistical power to detect smaller differences between methods. Second, only one IOS and one spectrophotometric device were evaluated; the findings cannot necessarily be generalized to other brands or generations of scanners, which may use different calibration and shade‐mapping algorithms. Third, the analysis focused on categorical value levels rather than instrumental color differences (Δ*E*). While this choice reflects how clinicians actually use shade guides, it prevents direct numerical comparison with studies that rely solely on Δ*E* thresholds. Fourth, although each measurement was repeated to reduce random error, the study did not compute specific repeatability or reproducibility indices for the scanner or the observers. Fifth, natural daylight may vary in intensity and color temperature despite time standardization. Despite these limitations, the present findings provide clinically relevant information about how an IOS and trained observers behave when evaluated on the value component against a spectrophotometric reference. Future research should expand the sample size, include additional scanner systems, and combine value analyses with Δ*E*‐based thresholds. Investigating training protocols that emphasize value recognition, as well as AI‐assisted systems that integrate full‐arch color and geometry data, may further enhance the reliability of shade selection.

### 4.8 Clinical Implications

Within the limitations of this study, several clinical implications can be drawn. First, low exact shade‐match percentages do not necessarily mean that digital or visual methods are clinically unreliable; when brightness is analyzed separately and a realistic ± 1 value level tolerance is allowed, both the scanner and trained observers achieve high levels of agreement with the spectrophotometric reference. Second, IOSs should be viewed as valuable partners to, rather than replacements for, visual shade selection. They offer objective, repeatable, and easily communicated data, but final verification by the clinician remains essential, especially in the anterior region. Third, both VITA Classical and VITA 3D‐Master can be used effectively for value assessment, although the Classical guide showed higher exact value agreement in this clinical model. Fourth, tooth type must be taken into account when planning esthetic restorations: posterior teeth appear more forgiving in terms of value, whereas maxillary incisors are the most demanding and require meticulous shade selection.

## 5 Conclusion

Within the limits of this clinical study, exact shade matching (hue–chroma–value) between the IOS (Medit i700), the spectrophotometric reference device (VITA Easyshade), and trained visual observers was generally low for both VITA Classical and VITA 3D‐Master. These findings indicate that the IOS, when used alone, is not sufficiently accurate for full shade selection in esthetic restorative dentistry. When the value component (brightness) was analyzed separately, agreement improved substantially. Both the scanner and the visual observers showed moderate rates of exact value match and high rates of value acceptability within ± 1 level of the reference value, suggesting that most mismatches occurred within a clinically tolerable range of brightness. The present results did not demonstrate a clear clinical advantage for either shade guide. Differences in value matching and value acceptability between VITA Classical and VITA 3D‐Master were small; VITA Classical showed a slightly higher exact value agreement than VITA 3D‐Master; however, value acceptability (± 1 level) was comparable between guides. Tooth type also influenced the behavior of value outcomes, with posterior teeth showing more stable agreement than anterior teeth, which is consistent with the higher translucency and more complex optical behavior of maxillary incisors. Overall, the Medit i700 scanner provided clinically acceptable performance for value determination but did not achieve sufficient accuracy in full shade matching to be used as a stand‐alone method. A multimodal approach that combines intraoral scanning with spectrophotometric measurements and controlled visual assessment remains advisable, particularly for highly demanding anterior restorations.

## Author Contributions

A.S.B. contributed to study design, data collection, analysis, and manuscript drafting. N.A. supervised the research, contributed to study design, and critically revised the manuscript.

## Funding

The authors have nothing to report.

## Conflicts of Interest

The authors declare no conflicts of interest.

## Data Availability

The data that support the findings of this study are available from the corresponding author upon reasonable request.
